# A Miniature Dual-Biomarker-Based Sensing and Conditioning Device for Closed-Loop DBS

**DOI:** 10.1109/JTEHM.2019.2937776

**Published:** 2019-08-30

**Authors:** Mahboubeh Parastarfeizabadi, Abbas Z. Kouzani

**Affiliations:** School of EngineeringDeakin University 2104 GeelongVIC3216Australia

**Keywords:** Analog circuit, brain sensor, deep brain stimulation, fabrication, multiple biomarkers

## Abstract

In this paper, a dual-biomarker-based neural sensing and conditioning device is proposed for closing the feedback loop in deep brain stimulation devices. The device explores both local field potentials (LFPs) and action potentials (APs) as measured biomarkers. It includes two channels, each having four main parts: (1) a pre-amplifier with built-in low-pass filter, (2) a ground shifting circuit, (3) an amplifier with low-pass function, and (4) a high-pass filter. The design specifications include miniature-size, light-weight, and 100 dB gain in the LFP and AP channels. This device has been validated through bench and in-vitro tests. The bench tests have been performed using different sinusoidal signals and pre-recorded neural signals. The in-vitro tests have been conducted in the saline solution that mimics the brain environment. The total weight of the device including a 3 V coin battery, and battery holder is 1.2 g. The diameter of the device is 11.2 mm. The device can be used to concurrently sense LFPs and APs for closing the feedback loop in closed-loop deep brain stimulation systems. It provides a tetherless head-mountable platform suitable for pre-clinical trials.

## Graphical abstract

This paper presents a miniature (radius: 5.6 mm), lightweight (total weight: 1.2 g) tetherless, and self-contained dual-biomarker-based neural recording device which is designed to be used in conjunction with a Deep Brain Stimulation (DBS) device to create a closed-loop function for the adjustment of stimulation parameters. Both local field potentials and action potentials are sensed and conditioned simultaneously as the biomarkers for DBS pulse adjustments. Results obtained through bench and in-vitro experiments confirm the recording capabilities of the designed neural recorder device. It provides a tetherless head-mountable platform suitable for pre-clinical trials.

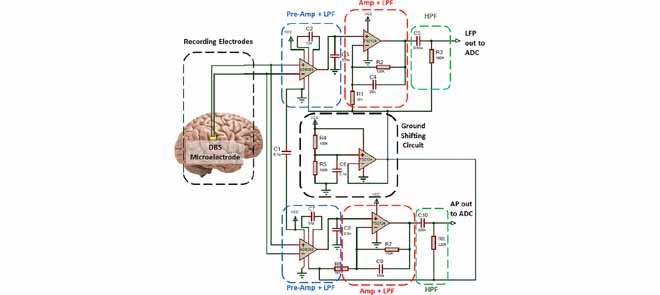

## I. Introduction

Deep brain stimulation (DBS) devices are classified into open-loop and closed-loop groups based on their internal function. In the open-loop DBS, non-stop stimulation pulses are delivered into the brain regardless of the variations in the brain’s condition. In the closed-loop DBS, on the other hand, the stimulation pulses are adjusted and delivered into the brain according to the variations in the brain’s condition.

Most of the current DBS systems operate in an open-loop manner. Open-loop DBS may however produce some significant functional (e.g. induction of paresthesia, involuntary movements, worsening of gait or speech, gaze deviation or paralysis), as well as cognitive, and mood side effects [1]. Closed-loop DBS can alleviate these side effects through optimization of the stimulation parameters (pulse width, amplitude, and frequency) [1], [2]. For this purpose, a biomarker is continuously measured and analyzed to optimize stimulation pulses according to the brain’s clinical condition. Therefore, the risks of the brain over- or under-stimulation can be minimized [3]–[6].

The existing closed-loop DBS devices [7]–[15] employ only one biomarker as the input to their control module to adjust the stimulation parameters. While some of these devices can be used to measure more than one biomarker (e.g., local field potentials (LFPs) and action potentials (APs)) [7]–[9], [13], [14], they have not implemented parallel acquisition of multiple biomarkers at the same time. Therefore, they optimize the stimulation parameters based on only one biomarker.

Closing the feedback loop based on one biomarker may not always ameliorate a spectrum of disease motor signs. For example, patients with Parkinson’s disease (PD) may experience different primary (e.g. tremor, bradykinesia, rigidity, postural instability, etc.) or secondary (e.g. freezing, micrographia, mask-like expression, unwanted accelerations) motor symptoms [16]. However, not all of these symptoms are promoted by the same pathophysiological neuronal systems. For example, rigidity and bradykinesia are functionally discrete from tremor [17]. Therefore, they may require separate neurophysiological biomarkers to adequately capture them [18]. Amelioration of a range of disease motor symptoms through only one biomarker could be a challenging task. Moreover, as discussed by Little and Brown [18], a single biomarker closed-loop control, may be efficient for tackling only some impairments. Therefore, in order to alleviate symptoms of a disease, a closed-loop system involving multiple biomarkers can perform better than one with only one biomarker [19].

**FIGURE 1.**
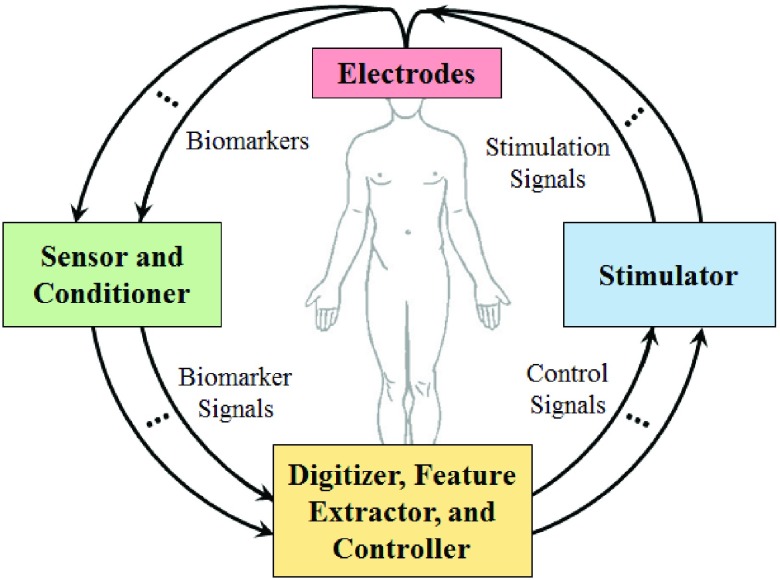
Multiple-biomarker-based closed-loop DBS architecture.

Fig. 1 shows a closed-loop DBS system involving multiple biomarkers. The overall system includes four components: (1) sensing/stimulating electrodes, (2) sensor and conditioner, (3) digitizer, feature extractor, and controller, and (4) stimulator. The first component includes sensing and stimulating electrodes. The sensing electrodes are used to read the biomarkers from the brain and other part of the body. The sensed biomarkers are amplified and filtered within the second component, sensor and conditioner, to produce biomarkers signals. The third component, digitizer-feature extractor-controller, samples the biomarkers signals, digitize them, and then extract key features out of them. The features are used as inputs to the controller which then forms control signals for modifying the stimulation parameters in the stimulator. The fourth component, stimulator, generates stimulation signals based on the input control signals. Finally, the stimulation signals are delivered to the brain via the stimulating electrodes.

Use of multiple biomarkers provide more information to the controller. Therefore, a more accurate assessment of the patient clinical state is carried out. Typical biomarkers that have been used in closed-loop DBS include surface electromyogram (sEMG), cortical neuro-signals such as electrocorticogram (ECoGs), subcortical neuro-signals such as LFPs (low-frequency oscillations) and APs (high-frequency oscillation), and neurochemical signals such as dopamine neurotransmitter release. A review by Arlotti *et al.* [20] concluded that, out of these biomarkers, LFP should be a primary choice because it contains more information needed for an ideal DBS biomarker. LFPs contain information about PD major symptoms such as bradkykinesia and rigidity. Besides, APs are considered to be a complementary biomarker for LFPs which together may enhance the outcome of DBS [21]. Johnson *et al.* [22] concluded that closed-loop systems that use multiple biomarkers, for example LFPs and APs, would provide more effective control in the system.

This paper focuses on measurement of two biomarkers, LFPs and APs, simultaneously. It presents a miniature dual-biomarker-based sensing and conditioning device. This device is portable and head-mountable, and will form the Sensor and Conditioner component of a closed DBS system. It will provide a great tool for use in tetherless head-mountable closed-loop DBS configurations for pre-clinical animal investigations.

## II. Methodology

### A. Action and Local Field Potentials

Any part of a neuron including soma, dendrites, axon, and axon terminals, contributes to the ionic processes in the extracellular space [23]. Extracellular potentials contain all the information from fast potentials to slow fluctuations [23]. The fast potentials are called extracellular APs, and the slow fluctuations are referred to as LFPs. APs include spikes and spike-induced after-hyperpolarization potentials. LFPs, on the other hand, are produced by spreading of APs through axons. High-frequency power of extracellular potentials provides indirect access to the intracellular APs [24], mainly indicating the spiking activities [23]. Hence, it is feasible to measure the extracellular APs from the same electrodes as LFPs.

It should be noted that LFPs and APs are not directly used as biomarkers by themselves. They contain several frequency bands, and activities within those bands are used as potential biomarkers for symptoms of neurological disorders. These frequency bands include alpha (8–14 Hz), beta (~13–30 Hz), slow-gamma (sG: 30–45 Hz), fast high-frequency oscillations (fHFOs: 300–400 Hz), and spikes (>500 Hz). Alpha has been shown to be high in the limbic system of major depressive disorder patients, correlating with severity symptom [25]. Similarly, it is shown to be maximal in pedunculopontine nucleus (PPN) region of PD patients, correlating with improved gait performance [26]. In this situation, a closed-loop DBS should work towards suppression of alpha biomarker. Beta, a well-known biomarker for PD [27], has prominent synchronization in the STN region of PD human and also animal models. A closed-loop DBS should work towards beta suppression, which causes improvement of rigidity, akinesia and bradykinesia [28], as well as the freezing of gait [29] symptoms in PD. In humans with Tourette’s syndrome, increased thalamic sG activity correlates with symptom relief following DBS [30]. In addition, suppression of sG oscillations is linked to DBS-induced tremor symptom reduction in PD patients [31]. In PD [32], [33] and epilepsy [34], [35], the existence of high-frequency oscillations (HFOs) is frequently detected. Furthermore, HFOs has been observed in the STNs of patients suffering from essential tremor and dystonia [36]. Specific changes in neuronal spike firing rate may be representative of seizure occurrence in epileptic patients [37], which could be used as a potential biomarker in a closed-loop DBS device.

LFPs and APs are typically weak bio-potentials and need amplification and filtering before being used in the feedback loop. The magnitude of these potentials can be in a range from $10~\mu \text{V}$ to 1 mV depending upon the electrode type and position [38], [39]. The frequency range of the LFPs is variable between 1 to 500 Hz [39], [40], while that of APs is much higher, and mainly changes between 300 Hz − 6 kHz [41].

### B. Circuit

**FIGURE 2.**
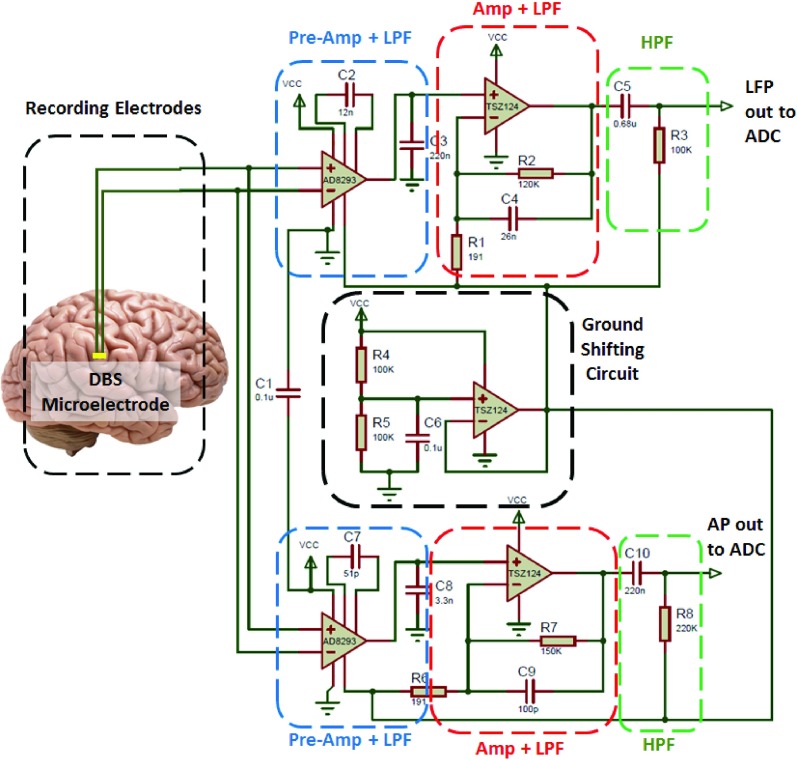
Front-end circuit diagram of the dual-biomarker-based sensing device. The LFPs and APs are differentially recorded from two contacts of a DBS microelectrode. The blue rectangles signify pre-amplifier and low-pass filter. The black rectangle demonstrates the ground shifting circuit. The red rectangles indicate the amplifier and low-pass filter. And, the green rectangle shows the high-pass filter circuit.

The dual-biomarker-based neural recording device was developed using discrete components to achieve higher flexibility in modifying the design, lower production expenses, and shorter manufacturing time, compared with application specific integrated circuits (ASICs) [42]. The designed device consists of four major parts: (1) a pre-amplifier combined with a low-pass filter, (2) an amplifier combined with a low-pass filter, (3) a high-pass filter, and (4) a ground shifting circuit. A schematic of the circuit is presented in Fig. 2.

This device has been specifically developed to be used in a closed-loop DBS system. It meets the DBS requirements on LFP and AP amplification and artifact rejection conditions. Rossi et al. [43] defined the requirements on LFPs as follows. A gain of 80 – 100 dB in the band-width of 2 – 40 Hz is needed to remove the 130 Hz (−40 dB) stimulation artifact from the LFP biomarker [43]. We have selected the requirements of the APs similar to those of LFPs but with the band-width of 300 Hz - 6 kHz.

Both the LFP and AP biomarkers can be differentially measured from a two-channel electrode as can be seen in Fig. 2. A low noise, low offset voltage, high common-mode rejection ratio (CMRR) precision instrumentation amplifier (IA), AD8293G160, was used in the pre-amplification stage of each channel. This IA has a fixed gain of 160 V/V. By means of two external capacitors (C2 and C3 in LFP and C7 and C8 in AP paths), the IA also implements a 2-pole low-pass filter. An internal 320 $\text{k}\Omega $ resistor forms a low-pass filter along with C2. Another 5 $\text{k}\Omega $ internal resistor forms a second low-pass filter together with C3. Based on the filter equations, C2 and C3 were chosen as 12 nF and 0.8 uF, respectively, implementing a 40 Hz LPF in the LFP channel. C7 and C8 capacitors were calculated 82 pF and 5.3 nF, respectively, to create a 6 kHz LPF in the AP path. To achieve optimum performance of the AD8293G160 chip, a $0.1~\mu \text{F}$ capacitor (C1 in Fig. 2) was connected between the supply (VCC) and the ground (GND) lines. This capacitor suppresses the excessive noises on the VCC pin and prevents undesired offset voltage in the output of the chip.

A ground shifting circuit is developed to provide the single-supply operation to the instrumentation and operational amplifiers, and to enable driving a single-supply analog-to-digital converter (ADC). This circuit shifts the ground to the mid-supply voltage. It comprises a voltage divider (R4, R5, and C6 in Fig. 2) followed by a unity gain buffer (TSZ124). This circuit drives both the LFP and AP paths. Due to the use of a supply voltage of 3 V from a CR1025 coin battery, the signals are shifted with an offset of 1.5 V using this technique.

Following the first stage, an amplifier with a single pole active non-inverting LPF was used to further reject the signal artifacts and boost the LFP and AP signals to a 105 V/V overall gain factor. A TSZ124, a very high accuracy quad operational amplifier chip, was used as the op-amps in the circuits of amplifier and low-pass filter, and ground shifting circuit. This chip benefits from a zero drift and micro-power properties to suit battery-operated experiments. In the final stage of the analog part, there is a first order passive HPF (with 2 Hz and 300 Hz cut-off frequencies for LFPs and APs, respectively) that removes the unwanted lower frequencies from the biomarkers.

Finally, the LFPs and APs can then be converted to digital values, through a single-supply, ADC for processing and adjustment of stimulation parameters.

### C. Fabrication

**FIGURE 3.**
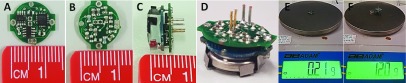
(A) Top view of the device. (B) Bottom view of the device. (C) Side view of the device. (D) Complete device. (E) Weight of the device without battery and battery holder. (F) Total weight of the complete device.

The neural recorder device was fabricated on a two-layer printed circuit board (PCB) with a circular configuration as shown in Fig. 3. In terms of the dimensions, this device benefits from a miniature size with a radius of 5.6 mm that enables head-mountable use with small laboratory animals such as mice. The complete device with battery, battery holder, and pins for connection to Plastic-One electrodes are shown in Fig. 3 (D). The weight of the device is only 0.21 g without the battery, 0.8 g with a CR1025 3 V coin battery, and 1.2 g with attached battery holder (see Fig. 3 (D) and (E)). To the best of our knowledge, this device is the lightest and smallest existing neural recorder designed with discrete components.

## III. Experimental Results

### A. Frequency Response

**FIGURE 4.**
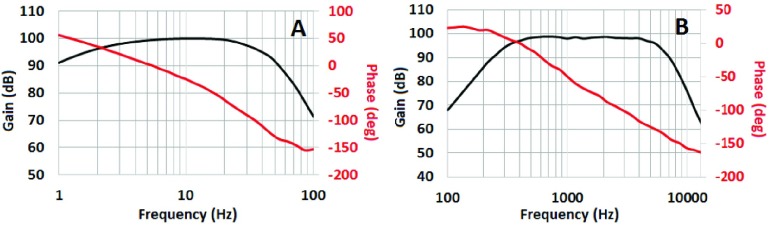
Bode plot showing gain and phase responses of the LFP (A) and AP (B) channels.

The bode plot showing the gain and phase responses of the LFP and AP channels is presented in Fig. 4. The data was captured by a NI myDAQ device through its bode-plot software, and then plotted within MATLAB software. As shown in Fig. 4, the device has a fixed gain of 100 dB in both the LFP and AP channels. The signal bandwidth is designed to be 2 – 40 Hz and 300 Hz – 6 kHz for the LFP and AP channels, respectively. The actual −3dB cut-off frequencies can be seen slightly affected due to the use of resistors and capacitors with 0.1–10% tolerances. The phase response of the LFP and AP channels are presented in red color in Fig. 4. The phase response changes from roughly 100° to 60°, and 100° to 50° as the frequency is swept from 1 to 100 Hz and 100 to 10000 Hz in the LFP and AP channels, respectively. The phase response showing a positive phase shift in the lower frequencies and a negative phase shift in higher frequencies (+50 to −150). However, it smoothly changes from positive to the negative values with no fast variations, which indicates the stability of the designed device over different frequency ranges.

### B. Bench-Test Validation

**FIGURE 5.**
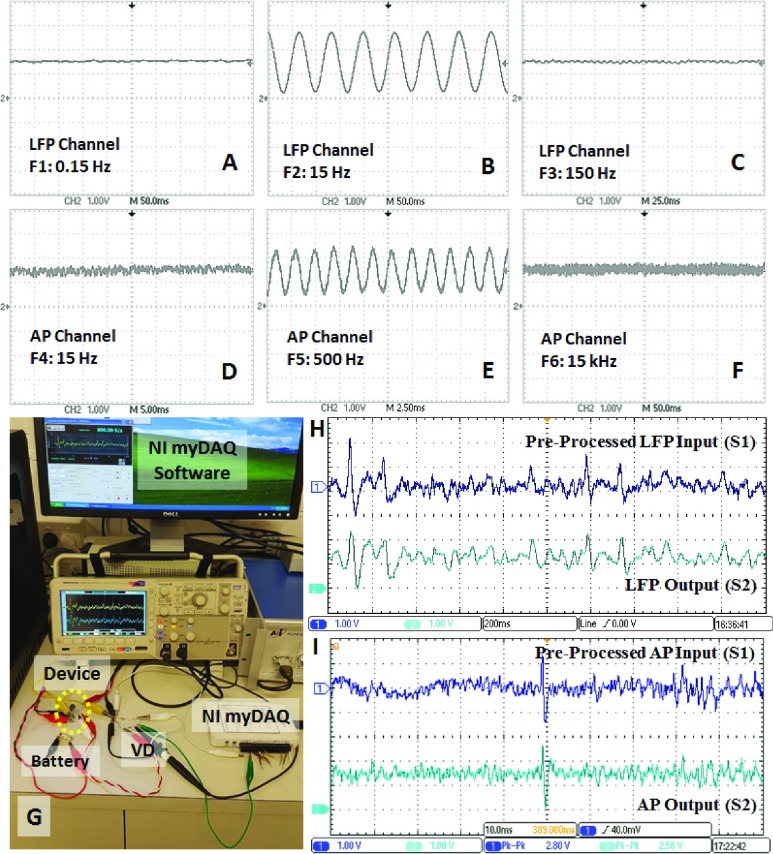
(A-F) The bench test outputs obtained from the LFP and AP channels for sinusoidal input signals. (G) Implemented bench test setup with pre-recorded neural signals. (H) LFP input applied to the voltage divider (VD), and the observed output from the LFP OUT pin. (F) AP input applied to the voltage divider and the observed output from the AP-OUT pin.

The functionality of the device was validated through applying sinusoidal and pre-recorded neural signals. Three sinusoidal signals with $25~\mu \text{v}$ amplitude and different frequencies (F1: 0.15 Hz, F2: 15 Hz, and F3: 150 Hz for LFP channel; F4: 15 Hz, F5: 500 Hz, and F6: 15 kHz for AP channel) were applied to the input of neural recorder using a signal generator. The F1 and F3 were chosen out of the low-pass and high-pass cut-off frequencies of the LFP channel. Similarly, F4 and F6 were chosen out of the working frequencies of the AP channel. To produce a $25~\mu \text{v}$ sine voltage, two voltage dividers were used at the input of each differential electrode. These voltage dividers were used to convert a 2 V AC signal to $45~\mu \text{V}$ and $20~\mu \text{V}$ AC signals for presenting to positive and negative inputs of the device, respectively. Hence, the device measured a $25~\mu \text{V}$ differential voltage (VIN+ - VIN−) at its input, and amplified it with a gain of 100,000 V/V. The observed outputs are presented in Fig 5 (A-F). As can be seen from Fig. 5 (A-F), the F1, F3, F4 and F6 were filtered by the device due to being out of the neural recorders’ working area. Only the F2 and F5 were well amplified.

Next, the procedure was repeated with pre-recorded neural data. The data was provided by Prof. Benoit Gosselin and Dr. Masoud Rezaei from Laval University, Canada. These signals were recorded from a 23 g mouse from the hippocampus area using a probe with approximately 8 $\text{M}\Omega $ resistance. The pre-recorded neural signal was introduced to the inputs of the device through the arbitrary waveform generator (ARB) of NI myDAQ system (via the analog output (AO-0)). The bench test setup and results are shown in Fig. 5 (G-I). The raw neural signal contained mainly high-frequency components recorded with a sampling frequency of 20 kS/s. The NI myDAQ software provides the capability of sending a signal with other sampling rates. Using this feature, high-frequency signals can be presented as signals whose frequency has been reduced. Therefore, to verify the LFP channel, we set the sampling frequency to 600 S/s to create a neural signal corresponding to a LFP signal. On other hand, to verify the AP channel, the pre-recorded neural signal was injected into the device with its original sampling frequency of 20 kS/s. Since the NI myDAQ device is not able to send low-amplitude potentials, we first amplified the original signal in MATLAB. Then, we used two different voltage dividers (2.2 $\text{M}\Omega $ with 22 $\Omega $, and 2.2 $\text{M}\Omega $ with $48~\Omega$) at the output of the NI myDAQ system to reduce the amplitude of the signal to $\mu \text{V}$ level for driving the In− and In+ inputs of the neural recorder device. The result of the LFP channel for the first 2 s is shown in Fig. 5 (H). The output has less variations to the input and well mimics the pattern. Fig. 5 (I) shows 100 ms of the input signal (S1) injected to the voltage dividers, and also the obtained output from the AP-OUT pin. As can be seen from the first 50 ms, the S1 contains low frequency variations which have been combined with the high-frequency components. These low-frequency variations do not appear at the output of the AP channel. Both the LFP and AP outputs are cantered at 1.5 V with over 2.5 Vpk-pk amplified voltage.

### C. In-Vitro Validation

**FIGURE 6.**
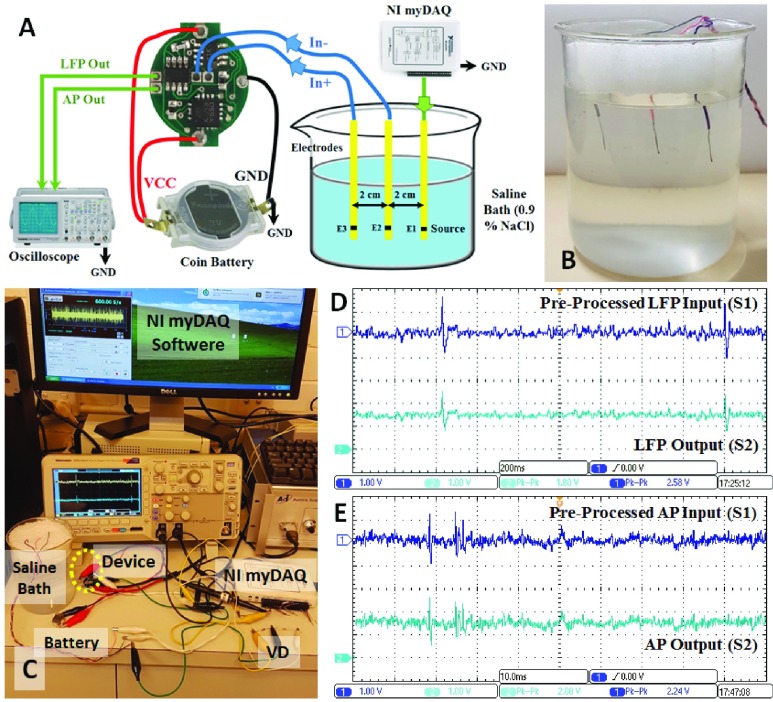
(A) In-vitro setup used for measurements in the saline solution. (B) Saline bath and copper electrodes used in the experiments. (C) Implemented in-vitro experimental setup. (D) LFP input to the voltage divider (VD) and the observed output from the LFP OUT pin. (E) AP input to the VD and the observed output from the AP-OUT pin.

The purpose of the in-vitro tests is to assess the sensing and conditioning abilities of the device in an environment that simulate a neuron-electrode interface in a neural recording system [44], [45]. The in-vitro setting includes a saline solution bath (0.9 % NaCl) prepared in the following steps: (1) solving of nine grams NaCl in one litter of distilled water, (2) gentle stirring of the solution for about ten seconds, (3) boiling of the solution for fifteen minutes, and (4) allowing the solution to reach the room temperature. Fig. 6 (A-B) illustrates the setup used for the in-vitro experiments. A copper electrode (E1) delivers the original pre-recorded neural signal received from the NI myDAQ system into the saline bath. This signal then propagates in different directions within the solution. Two other copper electrodes (E2 and E3) are placed near E1 to capture the signals propagating in the solution [46]. The differentially captured signal on E2 and E3 is then amplified and filtered by the device and the LFP and AP outputs are displayed on an oscilloscope for verification.

In order to verify the LFP channel, we set the sampling frequency of the original neural signal to 600 S/s to create a signal presenting LFPs. On other hand, to verify the AP channel, the pre-recorded neural signal was injected into the saline solution with its original sampling frequency of 20 kS/s. Due to the inability of the NI myDAQ system to output signals in $\mu \text{V}$ level, one voltage divider was used at its output (AO-0) to provide the saline bath with the potential in $\mu \text{V}$ level. Fig. 6 (C-E) represents the in-vitro setup and the results obtained for the LFP and AP channels. The results demonstrate the correct functionality of the device. As can be seen from Fig. 6 (D-E), the output follows the input pattern and is centered at the mid-supply voltage.

## IV. Discussions

This paper presents a dual-biomarker based neural recording device designed for concurrent LFP and AP measurements. The LFP channel amplifies the neural signals in the range of 2 – 40 Hz, and the AP channel boosts the signals in the range of 300 – 6000 Hz. A recent review by Amon and Alesch [47] discusses the recent advances and technical features of the electrodes and systems designed for DBS. Different electrode types are manufactured by three large companies including: (1) Medtronic, (2) Boston Scientific, and (3) St Jude. These electrodes have a contact length of 1.5–3 mm [47]. DBS-style macro-electrodes can be used to record LFPs. However, for measurements of APs, their contact length is orders of magnitude larger than a typical microelectrode, and thus average the signals from a much larger area around the contact. Plastics One electrodes, with a bare electrode diameter of 75–$250~\mu \text{m}$, have been developed for both electrophysiological recordings and electrical stimulations. The very small surface contact area of this electrode tip (0.004–0.049 mm2, versus ~6 mm2 in 3389 Medtronic DBS lead) will allow the extracellular APs to be collected from a much smaller area around the tip contact. In addition, this device is intended to record LFPs and APs in small animal primates (not humans). Usually in the laboratory animals because of several reasons (small brain size, costs, etc.), the use of Medtronic DBS lead (or other similar electrodes from other companies) is not preferred. In this case, usually microelectrodes or other electrodes with lower surface contact are desired. Plastics One electrodes have been frequently used in animal-based DBS experiments [48].

Due to the delivery of DBS [43], the 130 Hz artifact of DBS must be rejected. It is recommended in the literature that the artifact to be rejected in hardware instead of software [43]. Software-based post-filtering [49] degrades the signal quality due to the fact that neural signal amplitude is approximately five to six times smaller than that of the DBS artifact [43]. Therefore, manufacturing of a device with two separate channels for each of LFPs and APs are preferred over a 1-channel broadband neural recording device, despite the additional space and power consumption required.

This device benefits from a miniature size (only 11.2 mm in diameter) and a light weight (only 1.2 g including battery and battery holder). These features enable head-mountable closed-loop DBS investigations on small laboratory animals. The device is battery operated and consumes 2 mA current from a 3 V (30 mAh) lithium manganese dioxide coin battery (CR1025). Hence, the battery will last for 15 hours considering the collective 2 mA current requirement of the two channels.

One of the factors worth considering is the impedance of the recording electrodes in animal or human tissues. Higher electrode impedances can cause larger signal distortions at the input of the device [50]. Clinical impedance measurements of Plastics One electrodes show a range of 5 to 20 $\text{K}\Omega $ variations [51]. The impedance of this electrodes is considered to be low enough to prevent signal distortions. In addition, the high CMRR (140 dB) precision instrumentation amplifier, AD8293G160, at the input of our neural recorder can handle signal distortions. This electrode, as recommended in the manufacturer website, is a suitable option for both electrophysiological recordings and electrical stimulations.

The phase response of the device in LFP and AP channels is shown in Fig. 4 (A) and (B), respectively. The direction of the phase shift (whether it is positive or negative) is usually determined by the type of the filters [52], where the low-pass filters produce a negative phase shift (lag-response of the output in relation to the input) and high-pass filters produce a positive phase shift (lead-response of the output in relation to the input). The phase shift of the current device ranges from near +50 to −150 degrees over different frequency ranges because of the combination of low-pass and high-pass filters. However, it smoothly changes from positive to negative values with no fast variations in between, which shows the stability of the device over different frequency ranges. In a future application of the current neural recorder device in a closed-loop DBS paradigm, the phase shifts of the signals could be corrected if some temporal events in the brain (e.g. event based potentials) are considered as features to be extracted from the biomarkers [53].

This neural recording device has been designed using discrete components rather than ASICs. Therefore, we compare its features against discrete components based devices. Although ASICs may benefit from a better performance in terms of size and power consumption, they suffer from low flexibility and adaptability, extended design time and large prototyping costs [42]. In addition, ASICs are more appropriate for human-based long-term experiments rather than animal-based short-term trials. In the animal-based short-term trials, lower expenses and shorter design times are desirable [42], which can be provided by discrete-components-based design.

Our device can provide simultaneous dual-biomarker-based (LFP and AP) recordings, while most of the previous devices [10], [54]–[57] only record one biomarker at the same time. Although the gain and bandwidth of the current device is fixed and non-adjustable compared to the devices reported in ref. [54], [56], [57], our device benefits from a gain of 100 dB which is the highest gain achieved amongst the existing devices with a gain values between 35–100 dB. Moreover, in terms of the operating power supply, this device requires lower power voltage (3 V) compared to most of the existing devices with a minimum of 3.3 – 4 V power supply requirement [54]–[57]. The requirement for higher supply voltages results in the use of larger and heavier batteries, which can be avoided in our design because of lower supply requirements.

In terms of the power consumption, the neural recorder by Irwin et al. [56] has the lowest power consumption per channel ($681~\mu \text{W}$). Our neural recorder consumes 3 mW power per channel from a small 3 V coin battery. In addition, it has a reasonable battery lifetime of 15 hours for a 30 mAh capacity battery. Use of larger capacity batteries will increase the operation time. For example, a 3 V 1120 mAh lithium-ion battery (similar to chestek et al. work [55]), would theoretically provide over 23 days of continuous neural recording with our device.

This device benefits from a miniature physical size (11.2 mm in diameter, 0.6 cm in thickness) and is light in weight (1.2 g including battery and battery holder). Although this device might not be the smallest and lightest neural recorder, it is small and light enough to facilitate tetherless head-mountable battery-operated recording sessions on small freely-moving laboratory animals.

This neural recording device has been designed using discrete components rather than ASICs. Designing based on ASICs can help further reduce the device size and also minimize power consumption. Therefore, it is suggested as a future direction to design a multi-biomarker based neural recording device based on ASICs.

## V. Conclusion

This paper presented a miniature (radius: 5.6 mm), light-weight (total weight: 1.2 g) tetherless, and self-contained dual-biomarker-based neural recording device. It was designed to be used in conjunction with a DBS device to create a closed-loop function for the adjustment of the stimulation parameters. LFPs and APs are sensed and conditioned simultaneously as the biomarkers for DBS pulse adjustments. This device performance was assessed through both bench and in-vitro tests. The bench tests were conducted using sinusoidal signals and pre-recorded neural signals. The in-vitro assessments were performed in the saline solution which is a brain environment simulator. The results obtained through the bench and in-vitro experiments confirmed the recording capabilities of the designed neural recorder device.
